# Frequency and Significance of Body Weight Loss During Immunochemotherapy in Patients with Advanced Non-Small Cell Lung Cancer

**DOI:** 10.3390/cancers16234089

**Published:** 2024-12-06

**Authors:** Masataka Taoka, Eiki Ichihara, Toshihide Yokoyama, Koji Inoue, Tomoki Tamura, Akiko Sato, Naohiro Oda, Hirohisa Kano, Kayo Nakamura, Haruyuki Kawai, Masaaki Inoue, Nobuaki Ochi, Nobukazu Fujimoto, Hirohisa Ichikawa, Chihiro Ando, Isao Oze, Katsuyuki Kiura, Yoshinobu Maeda, Katsuyuki Hotta

**Affiliations:** 1 Department of Hematology and Oncology, Okayama University Graduate School of Medicine, Dentistry, and Pharmaceutical Sciences, Okayama 700-8558, Japan; po7a4c54@s.okayama-u.ac.jp (M.T.); kkiura@md.okayama-u.ac.jp (K.K.); yosmaeda@md.okayama-u.ac.jp (Y.M.); 2 Center for Clinical Oncology, Okayama University Hospital, Okayama 700-8558, Japan; 3 Department of Respiratory Medicine, Ohara Healthcare Foundation, Kurashiki Central Hospital, Kurashiki 710-8602, Japan; ty14401@kchnet.or.jp; 4 Department of Respiratory Medicine, Ehime Prefectural Central Hospital, Ehime 790-0024, Japan; c-koinoue@eph.pref.ehime.jp; 5 Department of Respiratory Medicine, NHO Iwakuni Clinical Center, Iwakuni 740-8510, Japan; doublemoon_season@hotmail.com; 6 Department of Internal Medicine, National Hospital Organization Okayama Medical Center, Okayama 701-1192, Japan; ahisamoto@gmail.com; 7 Department of Respiratory Medicine, Fukuyama City Hospital, Fukuyama 721-0971, Japan; dancingqueen121212@gmail.com; 8 Department of Respiratory Medicine, Japanese Red Cross Okayama Hospital, Okayama 700-0941, Japan; kotobukikaho.hiro@gmail.com (H.K.); orihihca@yahoo.co.jp (C.A.); 9 Department of Respiratory Medicine, Japanese Red Cross Himeji Hospital, Himeji 670-8540, Japan; kayo.nakamura.yanase@gmail.com; 10 Department of Internal Medicine, Okayama Saiseikai General Hospital, Okayama 700-8511, Japan; ha-kawai@po.harenet.ne.jp; 11 Department of Chest Surgery, Shimonoseki City Hospital, Shimonoseki 750-0041, Japan; masaaki2010@gmail.com; 12 Department of General Internal Medicine 4, Kawasaki Medical School, Okayama 701-0192, Japan; placidus.aura@gmail.com; 13 Department of Respiratory Medicine, Okayama Rosai Hospital, Okayama 702-8055, Japan; nobufujimot@gmail.com; 14 Department of Respiratory Medicine, KKR Takamatsu Hospital, Takamatsu 760-0018, Japan; ichikawa@kkr-ta-hp.gr.jp; 15 Division of Cancer Information and Control, Aichi Cancer Center Research Institute, Nagoya 464-8681, Japan; i_oze@aichi-cc.jp; 16 Center for Innovative Clinical Medicine, Okayama University Hospital, Okayama 700-8558, Japan; khotta@okayama-u.ac.jp

**Keywords:** non-small cell lung cancer, body weight loss, immune checkpoint inhibitors, chemotherapy

## Abstract

**Background:** Limited data are available on the frequency and significance of body weight loss during cancer therapy. This study investigated the frequency of patients who experienced body weight loss during immune checkpoint inhibitor (ICI) plus chemotherapy for advanced non-small cell lung cancer (NSCLC) and the impact of weight loss on treatment outcomes. **Methods:** Using the clinical data of 370 patients with NSCLC who received a combination of ICI and chemotherapy at 13 institutions, this study investigated the frequency of body weight loss > 5% during treatment and determined the impact of body weight loss on patient outcomes. **Results:** Of the 370 included patients, 141 (38.1%) lost more than 5% of their body weight during ICI plus chemotherapy (WL group). The 2-month landmark analysis showed that patients who experienced body weight loss of >5% during treatment had worse overall survival (OS) and progression-free survival (PFS) than those who did not (OS 14.0 and 31.1 months in the WL non-WL groups, respectively, *p* < 0.001; PFS 6.8 and 10.9 months in the WL non-WL groups, respectively, *p* = 0.002). Furthermore, a negative impact of body weight loss on survival was observed even in those who had obesity (body mass index [BMI] ≥ 25.0) at the start of therapy (OS 12.8 and 25.4 months in the WL non-WL groups, respectively, *p* < 0.001; PFS 5.7 and 10.7 months in the WL non-WL groups, respectively, *p* = 0.038). **Conclusions:** In conclusion, weight loss of >5% during ICI plus chemotherapy negatively influenced patient outcomes. Further and broader studies should investigate the role of nutritional status, specifically weight change and nutritional support, in responsiveness to ICI plus chemotherapy.

## Simple Summary

This study examined the frequency and impact of body weight loss during combination therapy with immune checkpoint inhibitors (ICIs) and chemotherapy for advanced non-small cell lung cancer (NSCLC). Among 370 patients treated at 13 institutions, 38.1% experienced weight loss exceeding 5% (WL group) during therapy. A 2-month landmark analysis revealed that the WL group had significantly worse overall survival (OS) and progression-free survival (PFS) compared to those without substantial weight loss (OS: 14.0 vs. 31.1 months, *p* < 0.001; PFS: 6.8 vs. 10.9 months, *p* = 0.002). This negative impact of weight loss was observed even in patients with normal or high body weight at baseline. The findings indicate that weight loss > 5% during ICI and chemotherapy adversely affects treatment outcomes, underscoring the importance of monitoring and managing weight changes in patients with cancer undergoing this therapy.

## 1. Introduction

The development of immune checkpoint inhibitors (ICIs) has revolutionized cancer treatment, offering improved survival outcomes for various malignancies, including non-small cell lung cancer (NSCLC) [1]. Anti-programmed death receptor-1 (PD-1) and anti-programmed death ligand-1 (PD-L1) inhibitors, used alone or in combination with chemotherapy, are now the standard treatments for advanced NSCLC [2,3,4,5,6]. Despite these advancements, some patients do not benefit from ICIs owing to host factors. Cancer cachexia and sarcopenia, characterized by significant weight and muscle loss, are among the negative factors affecting the efficacy of ICI therapy and remain major factors that negatively influence patient outcomes, likely owing to their immunosuppressive effects. For example, many studies have shown that patients with NSCLC who experience weight loss prior to ICI therapy are at a higher risk of tumor progression and have worse overall survival (OS) than those without weight loss [7,8]. The effects of weight loss on cancer treatment are a critical clinical concern because they reflect the underlying metabolic dysregulation and can lead to reduced treatment efficacy. A recent study reported that weight loss at the initiation of pembrolizumab treatment in patients with advanced NSCLC was associated with increased catabolic activity, higher pembrolizumab clearance, and significantly shorter OS [7]. These findings highlighted the complex interplay between metabolic changes and drug metabolism during ICI therapy. Moreover, cancer-related cachexia and sarcopenia are closely linked to systemic inflammation and immune suppression, both of which may interfere with the efficacy of ICIs and chemotherapy [8,9,10].

Considerable attention has been paid to the prognostic impact of weight loss before treatment as a negative indicator of the efficacy of ICI therapy. However, there is limited understanding of the frequency and clinical significance of weight loss during ICI therapy [11]. Studies have demonstrated that weight loss during cancer treatment is common and can significantly worsen treatment outcomes [12]. However, most of these studies were conducted after the era of ICI therapy and focused on conventional chemotherapy or single-agent ICIs, leaving a gap in knowledge regarding body weight loss during ICI combination therapies.

This study addressed these gaps by investigating the frequency and clinical significance of body weight loss during combination therapy with ICIs and chemotherapy in patients with advanced NSCLC. By focusing on weight loss during treatment, this study aimed to provide insight into its potential as a prognostic indicator and its relationship with treatment outcomes, including OS and progression-free survival (PFS). The findings of this study may contribute to improving patient management strategies by highlighting the importance of monitoring and mitigating weight loss during therapy. Furthermore, this study adds to the growing body of evidence emphasizing the metabolic and immunological complexities associated with advanced cancer and its treatment.

## 2. Materials and Methods

### 2.1. Patients

This was a branch study analyzing data from the Okayama Lung Cancer Study Group-Immune Checkpoint Database, which contains the clinical data of consecutive patients with NSCLC who started first-line systemic therapy (except for molecular-targeted therapy) for advanced NSCLC at 13 institutions in Japan from December 2018 to December 2020 [13,14].

### 2.2. Outcome and Exposure

The main outcome measures in the analysis were OS and PFS. OS was defined as the period from the initiation of ICI therapy to death, whereas PFS was defined as the period from the initiation of ICI therapy to disease progression or death. A landmark analysis was performed to avoid immortal time bias, and the landmark time was set at 2 months because, in practice, the evaluation of the treatment effect is performed at 2 months. In the landmark analysis, patients with worsening or death events in the first 2 months were excluded. Significant weight loss was defined as >5%, based on the definition of cachexia [15]. Patients were defined as having weight loss if they lost ≥5% of their body weight according to their weight at the initiation of therapy.

### 2.3. Statistical Analysis

The Cox proportional hazards model was used to analyze possible factors affecting patient survival. The model variables included >5% weight loss during therapy, baseline body mass index (BMI), age, sex, performance status (PS), histology, and PD-L1 expression. In the main analysis, the overall population was divided into two groups: patients with >5% weight loss compared to body weight at the initiation of therapy during ICI plus chemotherapy (WL group) and those without >5% weight loss (non-WL group). Parametric data were compared between the two groups using the Student’s *t*-test, and Fisher’s exact test was used to compare nonparametric data. The significance level was set at *p* < 0.05. Analyses were conducted using Stata statistical software (version 18; Stata Corp LLC, College Station, TX, USA).

## 3. Results

### 3.1. More Than One-Third of the Patients Significantly Lost Weight During Immunochemotherapy

In total, 370 patients with available body weight data during ICI treatment plus chemotherapy were included in this study. Of the 370 patients, 141 (38.1%) lost >5% of their weight during ICI plus chemotherapy, excluding body weight loss due to tumors.

Progression: this study also investigated the proportion of patients who lost weight only among those with the best response to therapy: complete response (CR) or partial response (PR). Overall, 212 patients achieved CR or PR in response to ICI plus chemotherapy. Among the 212 patients responsive to ICI plus chemotherapy, 83 (39.2%) lost >5% of their weight during treatment. The proportion of patients with weight loss was similar for all patients and those who achieved CR or PR with ICI plus chemotherapy.

The characteristics of the WL and non-WL groups are presented in Table 1. Pre-treatment BMI was not significantly different between the groups (median BMI 22.8 kg/m^2^ in the WL group and 22.1 kg/m^2^ in the non-WL group, *p* = 0.168). The median age was significantly higher in the WL group (71 years) than in the non-WL group (69 years) (*p* = 0.014). No other differences were observed between the groups.

### 3.2. Body Weight Loss During Treatment Negatively Affected Outcomes of ICI Plus Chemotherapy

**Figure 1:**
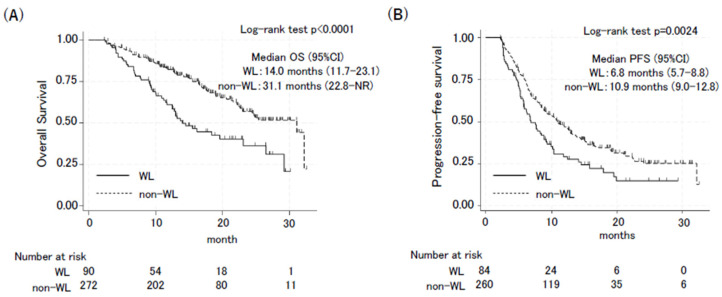
Kaplan–Meier curves of overall survival (**A**) and progression-free survival (**B**) in the WL group and non-WL group. OS and PFS between the WL group and non-WL group were compared using a 2-month landmark analysis. Confidence interval (CI).

The effects of body weight loss during treatment on patient outcomes were then determined. There were no significant differences in responses between the groups (WL vs. non-WL: disease control rate: 90.7% vs. 90.8%, *p* = 1.000; objective response rate: 58.7% vs. 56.3%, *p* = 0.666) (Table 2). The effect of body weight loss on survival outcomes was also assessed (Figure 1). Patients in the WL group had significantly worse PFS and OS than those in the non-WL group. The OS was 14.0 months in the WL group (95% confidence interval [CI]: 11.7–23.1) and 31.1 months in the non-WL group (95% CI: 22.8–not reached; hazard ratio [HR]: 2.18, 95% CI: 1.53–3.10, *p* < 0.001) (Figure 1A). The PFS was 6.8 months in the WL group (95% CI: 5.7–8.8) and 10.9 months in the non-WL group (95% CI: 9.0–12.8; HR: 1.56, 95% CI: 1.17–2.09, *p* = 0.003) (Figure 1B). The multivariable Cox regression analysis, including weight loss of >5% during the therapy, pre-treatment BMI, age, sex, PS, histology, and PD-L1 expression, showed that weight loss of >5% during the therapy was an independent poor factor for OS (HR 2.24, 95% CI: 1.51–3.36, *p* < 0.001) and PFS (HR 1.69, 95% CI: 1.20–2.36, *p* = 0.002) (Table 3).

### 3.3. Poor Prognosis Due to Weight Loss Was Also Observed in Patients with Standard Weight and Obesity

**Figure 2:**
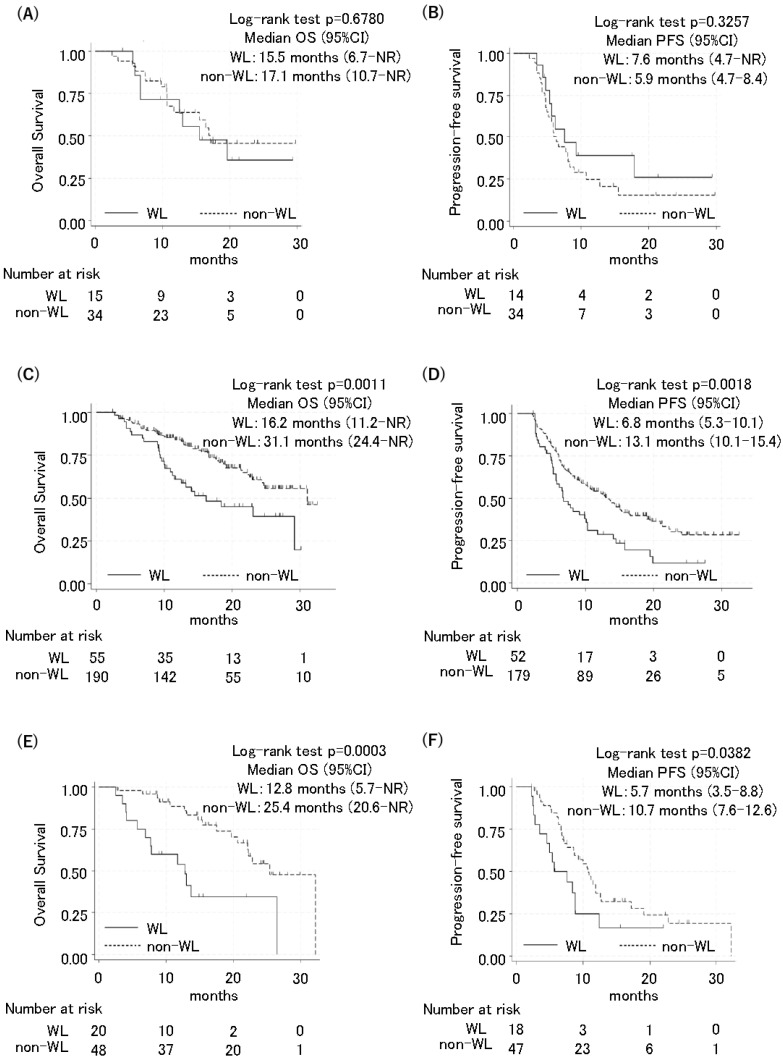
Kaplan–Meier curves of progression-free survival and overall survival for patients with low BMI, standard BMI, and high BMI. Overall survival and progression-free survival between the WL group and non-WL group were compared using a 2-month landmark analysis in patients with low BMI (<18.5) (**A**,**B**), standard BMI (18.5–24.9) (**C**,**D**), and high BMI (≥25) (**E**,**F**).

Finally, this study investigated whether the impact of body weight loss during therapy differed according to pre-treatment body weight status. The patients were then classified into low, standard, and high BMI groups using the indicators in Japan (low BMI: <18.5; standard BMI: ≥18.5 and <25; high BMI: ≥25.0) [15]. Among the patients with a low BMI, there was no difference in OS or PFS between the WL and non-WL groups. The OS was 15.5 months in the WL group [(95% CI: 6.7–not reached (NR)] and 17.1 months in the non-WL group (95% CI: 10.7–NR; HR: 0.68, 95% CI: 0.51–2.84, *p* = 0.679) (Figure 2A). The PFS was 7.6 months in the WL group (95% CI: 4.7–NR) and 5.9 months in the non-WL group (95% CI: 4.7–8.4; HR: 0.33, 95% CI: 0.32–1.47, *p* = 0.326) (Figure 2B).

Among the patients with a standard BMI, those in the WL group had a significantly shorter OS and PFS compared to those in the non-WL group (OS 16.2 months in the WL group [95% CI: 11.2–NR] and 31.1 months in the non-WL group (95% CI: 24.4–NR; HR: 2.09, 95% CI: 1.33–3.29, *p* = 0.001); and PFS 6.8 months in the WL group (95% CI: 5.3–10.1) and 13.1 months in the non-WL group (95% CI: 10.1–15.4; HR: 1.38, 95% CI: 1.23–2.56, *p* = 0.002) (Figure 2C,D). Similarly, among the patients with a high BMI, the WL group also had significantly shorter OS and PFS compared to the non-WL group. The OS was 12.8 months in the WL group (95% CI: 5.7–NR) and 25.4 months in the non-WL group (95% CI: 20.6–NR; HR: 3.74, 95% CI: 1.75–8.01, *p* < 0.001). The PFS 5.7 months in the WL group (95% CI: 3.5–8.8) and 10.7 months in the non-WL group (95% CI: 7.6–12.6; HR: 1.94, 95% CI: 1.02–3.66, *p* = 0.038) (Figure 2E,F).

### 3.4. Frequency of Most Immune-Related Adverse Events Was Not Different Between Groups

Furthermore, this study investigated the frequency of immune-related adverse events and compared the WL and non-WL groups (Table 4). There was no significant difference in the frequency.

## 4. Discussion

This study investigated the frequency of patients with advanced NSCLC who experienced body weight loss during ICI plus chemotherapy and examined the association between weight loss during therapy and survival outcomes. More than one-third of the patients lost more than 5% of their body weight during ICI plus chemotherapy, and poor OS and PFS were associated with weight loss.

Pre-treatment with low body weight or cachexia has been repeatedly reported to be associated with poor outcomes in patients with cancer [16,17,18]. Cachexia is a multifactorial syndrome frequently associated with cancer that includes loss of skeletal muscle, fatigue, functional impairment, decreased quality of life (QoL), and decreased survival, characterized by anorexia and unintended weight loss [8] and increased serum or tumor microenvironment concentrations of certain cytokines, such as tumor necrosis factor-α, interleukin (IL)-6, IL-8, and growth differentiation factor-15. It also increases the concentration of bone marrow-derived suppressor cells in plasma or tumor microenvironment [19]. Cachexia is also associated with immune system dysfunction and increased susceptibility to infection [20], both of which are presumed to weaken the efficacy of ICI therapy, leading to poor OS and PFS.

Weight loss often occurs during cancer pharmacotherapy [21]; however, the frequency of weight loss in patients with NSCLC receiving ICI plus chemotherapy remains unclear. Additionally, although many studies have focused on pre-treatment body weight status [22,23,24,25,26], no study has addressed the significance of body weight loss during therapy. Therefore, this study investigated these issues and, for the first time, found that 38.1% of patients experienced weight loss during ICI plus chemotherapy and that body weight loss during therapy was associated with poor outcomes.

Because it was previously reported that a lower pre-treatment BMI was associated with poor outcomes in ICI monotherapy [27], this study investigated the negative impact of body weight loss during therapy according to pre-treatment BMI. Interestingly, the impact of body weight loss during therapy differed according to the pre-treatment BMI. While the current study revealed that body weight loss during therapy led to inferior survival in patients with a pre-treatment standard or high BMI, such a negative effect was not observed in patients with a low BMI (Figure 2). Patients with a low BMI pre-treatment in the non-WL group showed numerically shorter survival than those with standard or high BMI (OS; 17.1 vs. 31.1 or 25.4 months. PFS; 5.9 vs. 13.1 or 10.7 months). The therapeutic effect in such patients may be likely already diminished, regardless of subsequent weight loss. However, it has been reported that the favorable prognosis associated with a high BMI is offset by the negative impact of weight loss before treatment [28], which is consistent with our current data. Collectively, these data suggest that even if a patient’s baseline weight is standard or higher, attention must be paid to the occurrence of weight loss.

However, the efficacy of nutritional interventions for weight loss in patients with cancer remains unclear [29]. Furthermore, whether preventing body weight loss during therapy through nutritional management improves the survival of patients with NSCLC remains unclear. There is evidence that nutritional support improves the QoL of patients receiving radiotherapy [30]; however, these results have not been confirmed in patients receiving pharmacotherapy [31]. Although little evidence is currently available to support the efficacy of nutritional interventions for patients with cancer, the European Society for Clinical Nutrition and Metabolism recommends identifying patients with cancer at nutritional risk through early screening, followed by nutritional counseling and nutritional support [32]. A multicenter randomized trial showed a reduced risk of short-term mortality and improved QoL with active nutritional support compared to usual hospital foods for patients with non-terminal cancer [33]. Further research is necessary to determine whether aggressive nutritional therapy improves the prognosis of patients with cancer treated with ICI plus chemotherapy.

This study had a few limitations. First, it was a retrospective cohort study with heterogeneous data. There were older patients in the WL group, which is potentially associated with poor prognosis. However, the multivariate analysis included age and showed that WL was an independent factor for poor survival. Secondly, there was no available data on body weight loss during chemotherapy or ICI therapy alone. Therefore, it remains unknown whether body weight loss during ICI plus chemotherapy is due to ICI treatment, chemotherapy, or both. Third, this study lacked data on weight loss before the initiation of ICI plus chemotherapy. Considering the possibility that there might have been more patients with cachexia who lost weight before the initiation of ICI plus chemotherapy in the WL group, the pre-treatment BMIs were compared between the WL and non-WL groups. No significant differences were observed between the two groups. Finally, this study did not reveal whether nutritional management during ICI plus chemotherapy improves patient survival. Further clinical studies are required to address this issue.

## 5. Conclusions

Patients with NSCLC who experienced weight loss during ICI plus chemotherapy had a shorter OS and PFS than those without weight loss. A 5% weight loss during ICI plus chemotherapy serves as an early indicator of suboptimal therapeutic response. These findings emphasize the importance of routine monitoring of body weight and nutritional status during treatment. Clinicians should consider incorporating nutritional assessments into standard care to effectively identify and manage at-risk patients. Additionally, future studies should explore the role of nutritional status, specifically weight change and nutritional support, in responsiveness to ICI plus chemotherapy.

## Figures and Tables

**Table 1:** Patient characteristics of the WL group and non-WL group.

	WL Group n = 141	Non-WL Group n = 229	
Median pre-treatment BMI (IQR)	22.8 (20.0–25.0)	22.1 (19.9–23.9)	*p* = 0.168
Median age, years	71 (40–83)	69 (34–84)	*p* = 0.014
Performance status			*p* = 0.086
0–1	124 (87.9%)	214 (93.4%)	
2–4	17 (22.1%)	15 (6.6%)	
Smoking history			*p* = 0.294
Yes	120 (85.1%)	186 (81.2%)	
No	19 (13.5%)	42 (18.3%)	
Unknown	2 (1.4%)	1 (0.5%)	
Sex			*p* = 0.691
Males	114 (80.9%)	180 (78.6%)	
Females	27 (19.1%)	49 (21.4%)	
Stage (rec/advanced/unknown)			*p* = 0.342
Advanced	114 (80.9%)	170 (74.2%)	
Postoperative recurrence	26 (18.4%)	57 (24.9%)	
Other	1 (0.7%)	2 (0.9%)	
Histology (non-sq/sq)			*p* = 0.455
Non-Sq	110 (78.0%)	170 (74.2%)	
Sq	31 (22.0%)	59 (25.8%)	
PD-L1 expression			*p* = 0.163
<50%	84 (59.6%)	149 (65.1%)	
≥50%	31 (22.0%)	54 (23.6%)	
unknown	26 18.4%)	26 (11.3%)	
EGFR/ALK mutation			*p* = 0.853
Yes	12 (8.5%)	22 (9.6%)	
No or undetermined	129 (91.5%)	207 (90.4%)	
Combination regimen			*p* = 0.887
Pembrolizumab-combined	118 (83.7%)	189 (82.5%)	
Atezolizumab-combined	23 (16.3%)	40 (17.5%)	

ALK, anaplastic lymphoma kinase; BMI, body mass index; EGFR, epidermal growth factor receptor; IQR, interquartile range; rec, postoperative recurrence; sq, squamous carcinoma; PD-L1, programmed death ligand 1; WL, >5% weight loss.

**Table 2:** Response outcomes to ICI plus chemotherapy in the WL and non-WL groups.

	WL Group n = 141	Non-WL Group n = 229	*p*-Value
Best response rate			
CR	1 (0.7%)	4 (1.7%)	
PR	82 (58.2%)	125 (54.6%)	
SD	45 (31.9%)	79 (34.5%)	
PD	11 (7.8%)	14 (6.1%)	
Unknown	2	7	
Disease control rate	128 (90.7%)	208 (90.8%)	*p* = 1.000
Objective response rate	83 (58.7%)	129 (56.3%)	*p* = 0.666

CR, complete response; ICI, immune checkpoint inhibitor; PD, progressive disease; PR, partial response; SD, stable disease; WL, weight loss > 5%.

**Table 3:** Multivariate analysis of the factor associated with progression-free survival (PFS) and overall survival (OS).

	PFS HR (95% CI)	*p*-Value	OS HR (95% CI)	*p*-Value
Body weight loss		0.002		<0.001
Yes	1.69 (1.20–2.36)		2.24 (1.51–3.36)	
No	1.0 (reference)		1.0 (reference)	

Model variables included weight loss of >5% during therapy, pre-treatment body mass index, age, sex, performance status, histology, and programmed death ligand expression. CI, confidence interval; HR, hazard ratio.

**Table 4:** irAEs in the WL and non-WL groups treated with ICI plus chemotherapy.

	WL Group n = 141	Non-WL Group n = 229	Fisher’s Exact Test
Any irAEs			
Any grade	59 (41.8%)	88 (38.4%)	*p* = 0.514
Grade 3 or more	24 (17.0%)	25 (10.9%)	*p* = 0.114
Hypothyroidism			
Any grade	6 (4.3%)	6 (2.6%)	*p* = 0.389
Grade 3 or more	0 (0%)	0 (0%)	
Pneumonitis			
Any grade	21 (14.9%)	33 (14.4%)	*p* = 0.881
Grade 3 or more	11 (7.8%)	12 (5.2%)	*p* = 0.377
Infusion reaction			
Any grade	0 (0%)	1 (0.4%)	
Grade 3 or more	0 (0%)	0 (0%)	
Colitis			
Any grade	5 (3.5%)	4 (1.7%)	*p* = 0.275
Grade 3 or more	2 (1.4%)	3 (1.3%)	*p* = 0.930
Hepatitis			
Any grade	1 (0.7%)	7 (3.1%)	*p* = 0.132
Grade 3 or more	1 (0.7%)	3 (1.3%)	*p* = 0.587
Skin reaction			
Any grade	8 (5.7%)	27 (11.8%)	*p* = 0.051
Grade 3 or more	1 (0.7%)	0 (0%)	
Hypophysitis			
Any grade	3 (2.1%)	2 (0.9%)	*p* = 0.310
Grade 3 or more	3 (2.1%)	2 (0.9%)	*p* = 0.310

ICI, immune checkpoint inhibitor; irAEs, immune-related adverse events; WL, weight loss > 5%.

## Data Availability

The datasets generated and/or analyzed in the current study are available from the corresponding author upon reasonable request.
